# Clinical and Biochemical Assessment of Liver Function Test and Its Correlation with Serum Ferritin Levels in Transfusion-dependent Thalassemia Patients

**DOI:** 10.7759/cureus.7574

**Published:** 2020-04-07

**Authors:** May Al-Moshary, Nayab Imtiaz, Eman Al-Mussaed, Adnan Khan, Samreen Ahmad, Sara Albqami

**Affiliations:** 1 Pathology, Princess Nourah Bint Abdul Rahman University, Riyadh, SAU; 2 Pathology, Institute of Kidney Disease, Peshawar, PAK; 3 Pediatrics, Rehman Medical Institute, Peshawar, PAK; 4 Internal Medicine, King Saud University, Riyadh, SAU

**Keywords:** thalassemia, bilirubin, alt, ast, ferritin

## Abstract

Aims

The aim of our study was to correlate liver function tests with serum ferritin levels in multi-transfused thalassemia patients.

Methods

This was a descriptive cross-sectional study conducted in the department of hematology, Khyber Medical University, from January 2018 to December 2018. Thalassemia patients of either sex dependent on transfusion ≥ 1 year and having a confirmatory report of the disease were included in our study. The nonprobability convenience sampling technique was used. The Pearson correlation coefficient was applied to observe the correlation between serum ferritin level and liver function tests. A p-value of ≤0.05 was considered statistically significant. SPSS version 23 (SPSS Inc., Chicago, Illinois) was used for data analysis.

Results

A total of 138 subjects of age range 2-23 years, with a mean age of 12.08 ± 6.02 years, were included in our study. The mean serum ferritin of patients in our study was 3278.64 ng/ml with the lowest of 285.2 ng/mL and the highest of 10940.2 ng/ml. With the increase in serum ferritin levels, a rapid increase in alanine aminotransferase (ALT), aspartate aminotransferase (AST), and alkaline phosphatase (ALP) levels was seen. When serum ferritin levels were correlated with total bilirubin level, the bilirubin level remains static with a further increase in serum ferritin levels.

Conclusion

It was deduced that iron deposition is the ultimate reason for increased liver enzymes. There was a positive correlation between serum ferritin and ALT, AST, and ALP while a weak connection was found between serum ferritin and bilirubin levels.

## Introduction

Thalassemia is derived from the Greek words, Thalas, which means sea, and emia, which means blood, signifying that it is more common in the Mediterranean region [1]. Globally, among humans, thalassemia is the commonest single-gene disorder. It is defined as a group of inherited disorders characterized by decreased or absent beta globin chain synthesis, leading to a reduced level of hemoglobulin (Hb) in the red blood cells [2]. Specifically in developing countries, thalassemia is a huge health dilemma.

Blood transfusion is the primary way of treating thalassemia; it allows the normal growth of the child as well as restrains abnormal erythropoiesis [3]. Iron-chelating agents should be used properly; otherwise, multiple blood transfusions can lead to iron overload. Yet, with no blood transfusion, the increase rate of erythropoiesis intensifies dietary iron absorption from the gut, leading to a severe form of iron overload [4]. iron overload can result in serious damage to various organs, for example, by depositing in the liver, heart, and various other endocrine glands along with endocrine organ failure. Cardiotoxicity is the most severe and life-threatening complication of iron overload, which needs chelation therapy [5].

The liver is the only site for ferritin and transferrin synthesis, as well as the primary organ for iron storage. Under normal conditions, iron is protein-bound in the liver and free ferrous iron is severely toxic. In unbound form, iron catalyzes the production of free radicals, which has been implicated in lipid peroxidation as well as in hepatotoxicity [6].

The liver has the maximum capacity to store excess iron in the body, and various other organs, as well as the liver, are very susceptible to damage as a result of iron toxicity. In other studies, the correlation between serum ferritin and hepatic iron concentration has been reported in multiple blood-transfused thalassemia patients [7]. However, there is a paucity of data regarding the correlation between iron overload and liver damage in thalassemic patients. Hence, the objective of this study was to correlate liver function tests with serum ferritin levels in multi-transfused thalassemia patients. The results of this study will provide us with local statistics, and this will open a window for further research.

## Materials and methods

This was a descriptive cross-sectional study conducted in the department of hematology, Khyber Medical University, between January and December 2018. A total of 138 patients were recruited by using the World Health Organization (WHO) formula for sample size estimation. Participant’s selection was done on the basis of non-probability convenient sampling. Transfusion-dependent thalassemia patients, ages between six and 20 years of either gender having a confirmatory lab report of Hb electrophoresis were included in the study. While individuals already having any disease (e.g., hepatitis and hemochromatosis) or having acute illnesses, such as fever and infections, were excluded from the study.

Before starting the study, institutional ethical approval was taken. Patients were selected for the study based on inclusion and exclusion criteria. Consent was received and signed from all patients. Information was gathered on a planned proforma. The initial segment of the proforma comprised details about the age, sex, name, and address of the patient while the second part incorporated clinical introduction and examination. Three ml of blood was taken from the cases by needle puncture of peripheral veins. The blood was centrifuged at 4000 rpm for 10 minutes in a centrifuge using the Yingtai TD4C instrument (Changsha Yingtai Instrument Co., Ltd., Hunan, China ). The serum thus obtained was taken in two test tubes, one to be tested and one to be stored for future purposes. The serum was stored in serum cups at -35 degrees centigrade in a refrigerator by (Sanyo Biomedical Freezer MDF-U537; Sanyo Electric Co., Ltd., Osaka, Japan).

The data were initially recorded on Microsoft Excel spreadsheets (Microsoft Corporation, Seattle, WA). Factual investigations were performed utilizing IBM SPSS (version 20) programming (SPSS Inc., Chicago, IL). Mean and standard deviation was determined for numerical factors. Frequencies and rates were determined for absolute factors. Age and sexual orientation shrewd stratification was done. Results were exhibited as tables and diagrams.

## Results

**Figure 1:**
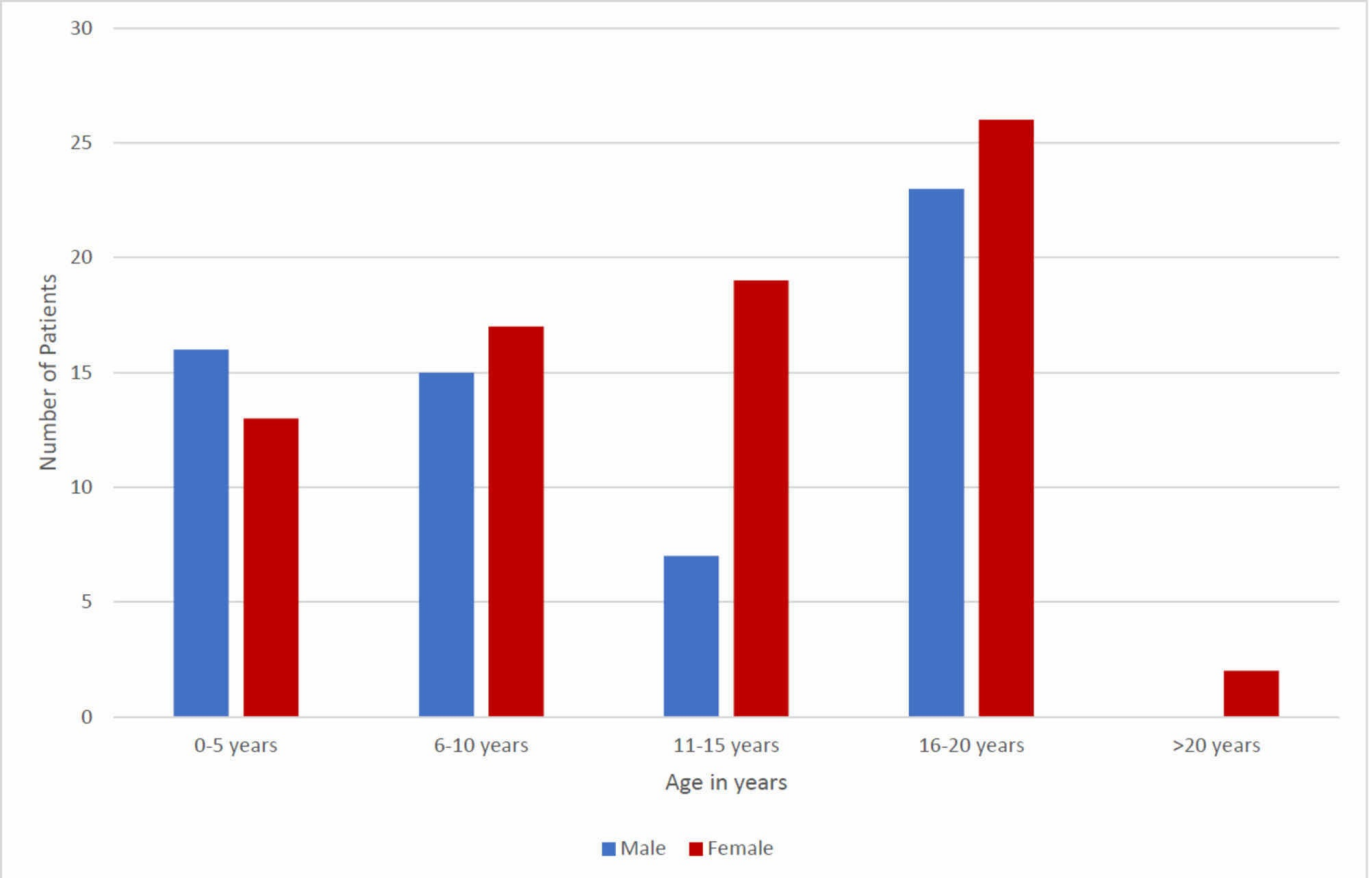
Age distribution (n=138)

A total of 138 patients with thalassemia were included in which 61(44.2%) were male and 77(55.8%) were female. The age range in our study was two to 23 years, with a mean of 11.86 ± 6.1 years. The mean age of male patients was 11.15 ± 5.65 while that of female patients was 12.42 ± 6.68. The majority of patients were in the age group 16-20 years (35.5%). The age distribution is shown in Figure 1.

The mean serum ferritin of patients in our study was 3172.044 ng/mL with a minimum of 285.2 ng/mL and a maximum of 10940.2 ng/ml. The mean alanine aminotransferase (ALT) level in our study was 37.10 IU/L with a standard deviation of ± 6.48, with the lowest of 02 IU/L and the highest of 143 IU/L. The rest of the parameters are shown in Table 1.

**Table 1:** Descriptive statistics of study parameters (N-138) ALT: alanine aminotransferase; AST: aspartate aminotransferase; ALP: alkaline phosphatase

Parameters	Serum ferritin (ng/mL)	ALT	AST	ALP	Bilirubin
Number of patients	138	138	138	138	138
Mean ( IU/L)	3172.044	37.109	90.1884	138.753	1.258
Std. Deviation	1748.1004	6.4875	11.59	20.93232	0.44
Minimum ( IU/L)	285.2	2.0	6.00	44.00	0.6
Maximum (IU/L)	10940.2	143.0	545.00	768.00	3.4

In our study, all male patients had a serum ferritin level of more than 300 ng/mL, with a mean of 2884.639 ± 1472.74, ranging from 349.9-5517 ng/ml. whereas in females, one patient had a serum ferritin level of 285.2 and the rest was more than 300, with a mean of 3399.61 ± 1917.65, ranging from 285.2 - 109440 ng/ml. The rest of the parameters are shown in Table 2.

**Table 2:** Distributive statistics of parameters according to gender ALT: alanine aminotransferase; AST: aspartate aminotransferase; ALP: alkaline phosphatase

Gender	Parameters	Mean	SD	Minimum	Maximum
Male	Serum ferritin	2884.639	1472.74	349.9	5517.2
	ALT	36.541	5.6447	13.0	140.0
	AST	83.1148	9.10804	12	345
	ALP	145.8525	12.11869	46	768
	Total bilirubin	1.321	0.4947	0.7	3.4
Female	Serum ferritin	3399.729	1917.6576	285.2	10940.2
	ALT	37.558	7.2954	2.0	143.0
	AST	95.7922	11.26114	5	545
	ALP	133.1299	16.33180	44	657
	Total bilirubin	1.208	.3936	0.6	2.5

**Figure 2:**
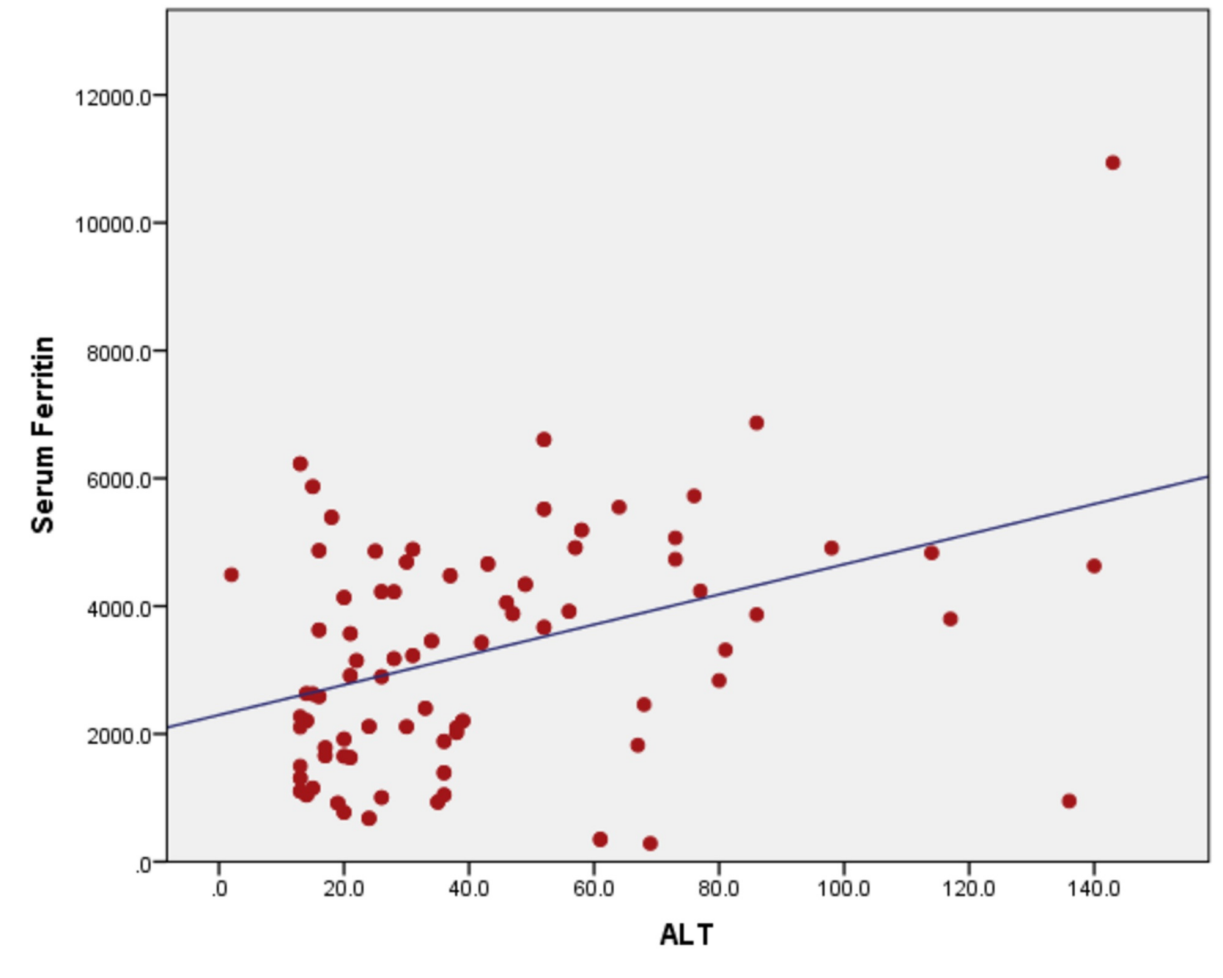
Correlation of serum ferritin with ALT (r = + 0.315) and a p-value of <0.001 ALT: alanine aminotransferase

**Figure 3:**
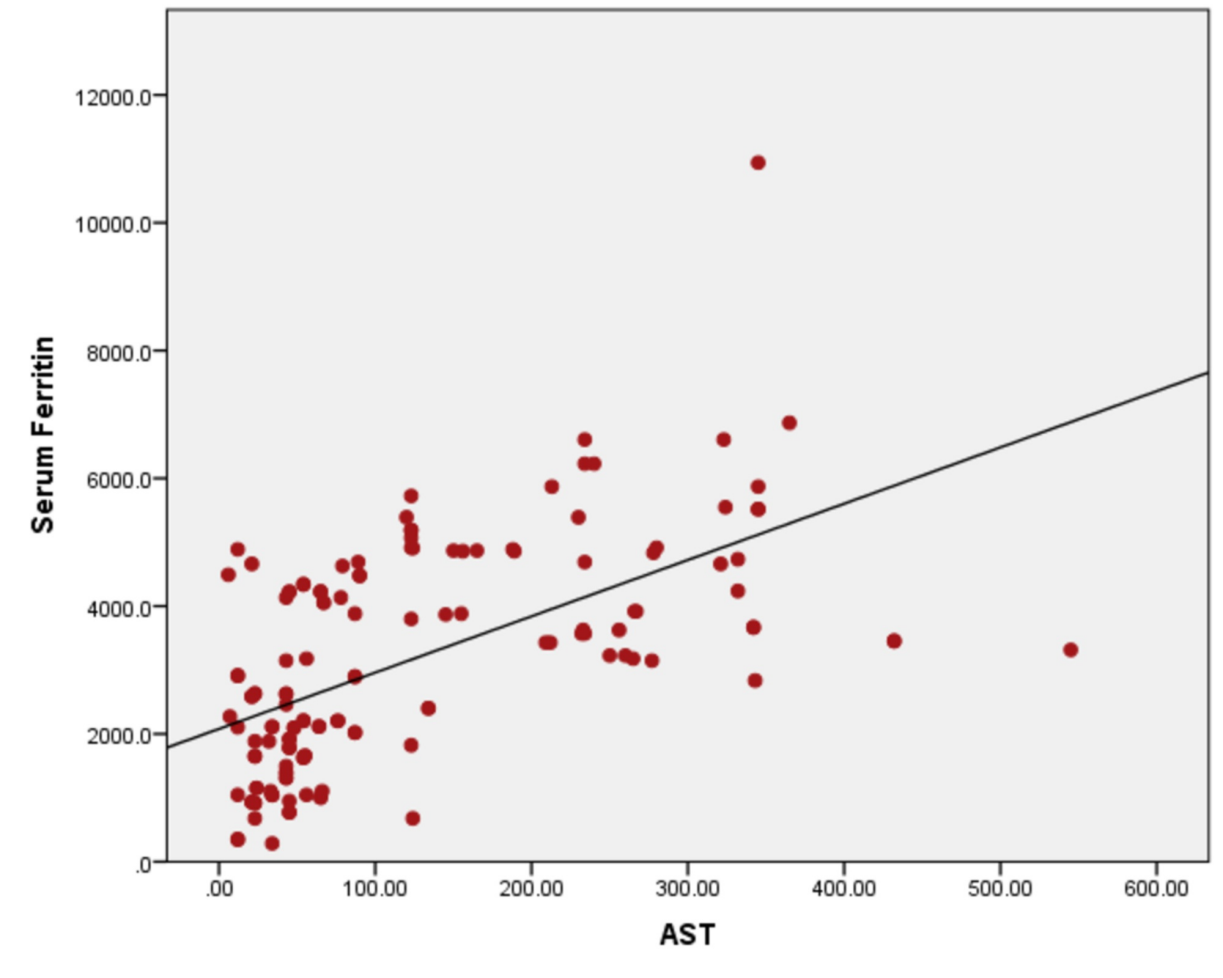
Correlation of serum ferritin with AST (r = + 0.670) and a p-value of <0.001 AST: aspartate aminotransferase

**Figure 4:**
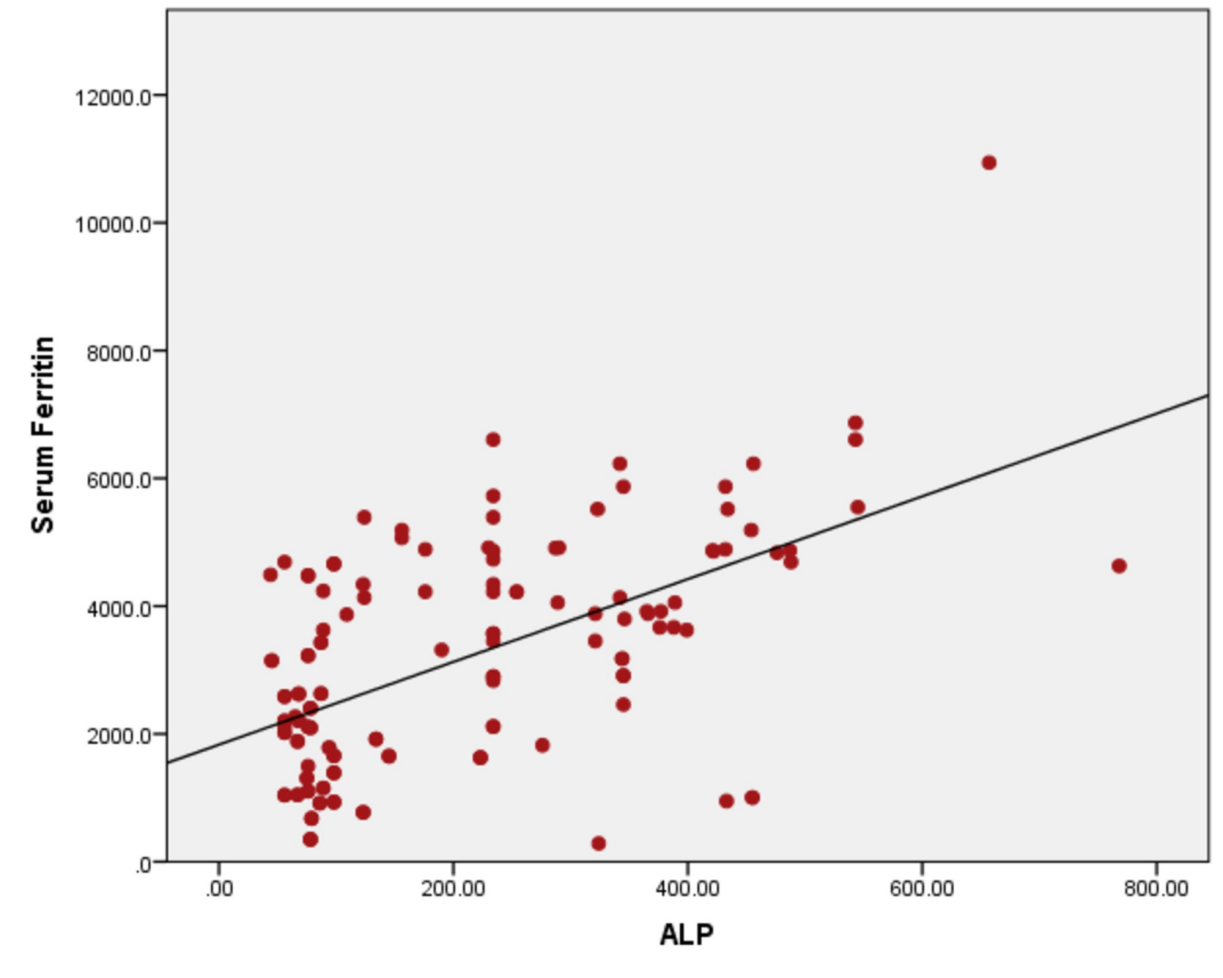
Correlation of serum ferritin with ALT (r= + 0.430) and a p-value of <0.001 ALT: alanine aminotransferase

**Figure 5:**
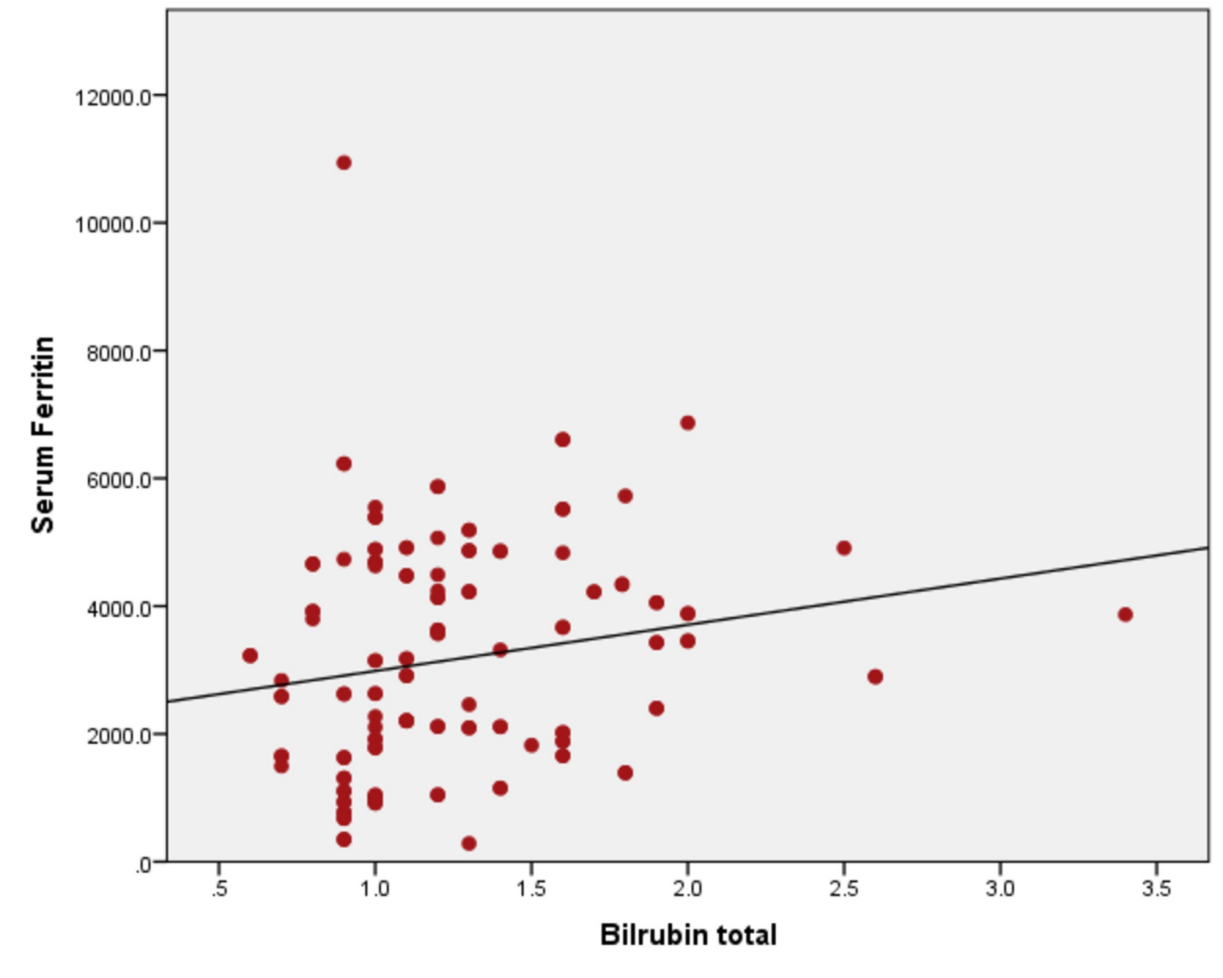
Correlation of serum ferritin with bilirubin (r= + 0.294) and a p-value of <0.001

When serum ferritin levels were correlated with ALT, the ALT levels remain in the normal range until serum ferritin levels of 1000 ng/ml. With a further increase in serum ferritin levels, the rapid increase in ALT levels was seen. The Pearson bivariate coefficient correlation was positive (r = + 0.319) with a p-value of <0.01. Figure 2 showing a correlation between ferritin and ALT by correlating serum ferritin with AST with r = + 0.670) and a p-value of <0.001, as shown in Figure 3. Ferritin vs ALP showed a positive correlation of r = + 0.430 and a p-value of <0.001 (Figure 4), with bilirubin r= + 0.294 and a p-value of <0.001, as shown in Figure 5.

## Discussion

Studies done in the past have shown some correlation between liver function tests (LFTs) and serum ferritin, but the results varied in most of the studies. Our study aimed to find the correlation of LFTs and higher levels of serum ferritin in local thalassemia patients who are on regular blood transfusion and poorly chelated. Limited research has been done on this perspective.

The cause of thalassemia is mainly mutations that change the state of at least one globin gene. To date, around 200 point mutations and a few deletions on chromosome 11 have been seen in the β-globin gene [8]. The iron deposits of the body are kept in a range of 200-1500 mg ordinarily in people with a normal iron deposition of 5 mg/kg in ladies and 13 mg/kg in men [9]. Every unit of blood has around 200 mg of iron, yet this dimension gradually and progressively raises by continuous blood transfusions in thalassemia patients [10].

To determine the toxic level of iron, the critical values of serum ferritin varies from 1000-3000 µg/L in different studies and the standard values of serum ferritin level have a very wide range in males (10-220 µg/L) and females (10-85 µg/L) in normal conditions [11]. Iron levels and serum ferritin have a positive correlation and because of this, the serum ferritin concentration is usually used to calculate the iron overload in β thalassemia patients [12].

In the current study, the average serum ferritin level (3278.64 ± 1862.33) was considerably higher than its peak value (1000 ng/mL), showing that regularly transfused patients are in iron overload status. Besides, a few investigations showed the abnormal state of ALT in thalassemia patients receiving various blood transfusions [13]. Thus, we have likewise discovered raised levels of ALT in the present investigation. There was a positive correlation between serum ferritin level and ALT in our study population (r = + 0.39) and a p-value of <0.01. Hence, the results of this study are similar to different studies. Consequently, the reason tends to be that the abnormal amounts of hepatic catalysts are potentially because of the hepatic damage caused by the iron overburden in thalassemia patients accepting a different blood transfusion. A few other researchers have a similar perspective with respect to the abnormal state of hepatic enzymes in thalassemia patients [14-15]. In the current study, we have found the elevated levels of AST, ALP, and serum bilirubin as well. The results of the present study are thus concurrent with the results of other studies done by De Sanctis et al. [13].

Some analysts have depicted the proposed component of activity, however, the correct process is not clear as yet. Seng Suk et al. found liver functions to be three- to four-folds increased in β-thalassemia patients than normal individuals [16]. The iron deposition is associated with increased oxidative stress, lipid peroxidation, and liver cell damage in transfusion-dependent β-thalassemia major. Jensen et al. also observed that serum transaminases and hepatic fibrosis increases as liver iron concentration increases [17].

The liver is the earliest site of iron deposition in regularly transfused thalassemia patients and a common cause of morbidity. Iron overload occurs both in hepatocytes and reticuloendothelial cells. Free radical production is increased in patients with iron overload through the Fenton reaction. These free radicals accumulate in the liver, heart, and other organs cause extensive tissue damage and play havoc [18].

## Conclusions

It was inferred that iron overburden is a principle driving reason for raised liver proteins and especially ALT, AST, and ALP. There was a positive correlation between serum ferritin and LFTs. Subsequently, further definite examinations ought to be led to investigate the real cause for this connection for this in the future and to discover the promising connections in thalassemia patients receiving numerous blood transfusions.
